# Professional Responsibility

**Published:** 1871-02

**Authors:** 


### Editorial.

### PROFESSIONAL RESPONSIBILITY.

> "The fruit
> Of that forbidden tree whose mortal taste
> Brought death into the world and all our woe,"

Though not now temptingly hanging on pendent boughs in the midst of Paradise, is, nevertheless, eaten by Eve's daughters and Adam's sons; and its mortal taste yet brings death and countless woes. And what a word is DEATH! It implies all the tendencies to and all the concomitants of dissolution. It means everything that is terrible! It includes all of sickness, sorrow, sadness, suffering and sighing. It suggests all the horrors of war, famine and pestilence. Its music is the widow's wail, the orphan's cry, the shriek of the assassin's victim, the maniac's wild laugh, and the groans of the wretched and miserable everywhere. Dynasties have gone down in ruins and mighty monarchs are dethroned; but Death, "the king of terrors," still sways his scepter over our beautiful world, and Nature is blasted by the blight of his countenance. No other sovereign has mustered such forces; nor has human king achieved such conquests. He leads the "destruction that wasteth at noon-day," and commands "the pestilence that walketh in darkness." The poisonous serpent and the more baneful cup are enlisted in his service; and debauchery, riot and murder extend his victories in all directions.

As a profession we have enlisted under the beautiful banner of human health, for a life-long warfare against the armies of this dread monarch; and it is time for us to fully appreciate the responsibilities which we have assumed. But it is to be feared that we are too much like the soldiers in the earlier struggles of our late civil war, when it was supposed that a prolonged pic-nic, varied with occasional militia musters, would answer all purposes, till the battle of Shiloh, unequaled, save in a single instance, since Waterloo, aroused all to the fact that both parties were in deadly earnest. If we realize the solemn, momentous truths, but feebly set forth in our first paragraph, we will either not enlist, or having enlisted, will prove worse than traitors, if we fail to give our best and holiest efforts to the cause. If our young men can be brought to a full recognition of such responsibilities, they will not rest satisfied with their attainments when they have learned to boil rubber or to press amalgam paste or putty into decayed dental organs as well as their preceptors. They will then feel that their professional knowledge must result in longer life, holier, purer, better, because healthier life, to the race of man. Members of our profession appreciating such truths, and feeling such responsibilities, will receive as students only such as can and will make the proper attainments, while they see that the best facilities for making such attainments are afforded. Till something like this has been reached, "our glorious profession" will be but little, if at all, better than a handicraft. W.

